# Pilot Study on High-Intensity Focused Ultrasound (HIFU) for Basal Cell Carcinoma: Effectiveness and Safety

**DOI:** 10.3390/jcm13113277

**Published:** 2024-06-01

**Authors:** Jacek Calik, Natalia Sauer, Bartosz Woźniak, Andrzej Wojnar, Paweł Pietkiewicz, Piotr Dzięgiel

**Affiliations:** 1 Department of Clinical Oncology, Wroclaw Medical University, 50-556 Wroclaw, Poland; 2 Old Town Clinic, 50-136 Wroclaw, Poland; barwoz91@gmail.com; 3 Department of Clinical Pharmacology, Faculty of Pharmacy, Wroclaw Medical University, 50-556 Wroclaw, Poland; 4 Department of Preclinical Sciences, Pharmacology and Medical Diagnostics, Wrocław University of Science and Technology, 50-370 Wrocław, Poland; 5 Zwierzyniecka Medical Center, Zwierzyniecka 30/28, 60-814 Poznań, Poland; pietkiewicz.pp@gmail.com; 6 Polish Dermatoscopy Group, 61-683 Poznan, Poland; 7 Division of Histology and Embryology, Department of Human Morphology and Embryology, Wroclaw Medical University, T. Chalubinskiego 6a, 50-368 Wroclaw, Poland; piotr.dziegiel@umw.edu.pl; 8 Department of Human Biology, Faculty of Physiotherapy, Wroclaw University of Health and Sport Sciences, 51-612 Wroclaw, Poland

**Keywords:** Basal Cell Carcinoma (BCC), High-Intensity Focused Ultrasound (HIFU), dermoscopic analysis, non-surgical skin cancer treatment

## Abstract

**Background:** The rising incidence of Basal Cell Carcinoma (BCC), especially among individuals with significant sun exposure, underscores the need for effective and minimally invasive treatment alternatives. Traditional surgical approaches, while effective, often result in notable cosmetic and functional limitations, particularly for lesions located on the face. This study explores High-Intensity Focused Ultrasound (HIFU) as a promising, non-invasive treatment option that aims to overcome these challenges, potentially revolutionizing BCC treatment by offering a balance between efficacy and cosmetic outcomes. **Methods:** Our investigation enrolled 8 patients, presenting a total of 15 BCC lesions, treated with a 20 MHz HIFU device. The selection of treatment parameters was precise, utilizing probe depths from 0.8 mm to 2.3 mm and energy settings ranging from 0.7 to 1.3 Joules (J) per pulse, determined by the lesion’s infiltration depth as assessed via pre-procedure ultrasonography. A key component of our methodology included dermatoscopic monitoring, which allowed for detailed observation of the lesions’ response to treatment over time. Patient-reported outcomes and satisfaction levels were systematically recorded, providing insights into the comparative advantages of HIFU. **Results:** Initial responses after HIFU treatment included whitening and edema, indicative of successful lesion ablation. Early post-treatment observations revealed minimal discomfort and quick recovery, with crust formation resolving within two weeks for most lesions. Over a period of three to six months, patients reported significant improvement, with lesions becoming lighter and blending into the surrounding skin, demonstrating effective and aesthetically pleasing outcomes. Patient satisfaction surveys conducted six months post-treatment revealed high levels of satisfaction, with 75% of participants reporting very high satisfaction due to minimal scarring and the non-invasive nature of the procedure. No recurrences of BCC were noted, attesting to the efficacy of HIFU as a treatment option. **Conclusions:** The findings from this study confirm that based on dermoscopy analysis, HIFU is a highly effective and patient-preferred non-invasive treatment modality for Basal Cell Carcinoma. HIFU offers a promising alternative to traditional surgical and non-surgical treatments, reducing the cosmetic and functional repercussions associated with BCC management. Given its efficacy, safety, and favorable patient satisfaction scores, HIFU warrants further investigation and consideration for broader clinical application in the treatment of BCC, potentially setting a new standard in dermatologic oncology care. This work represents a pilot study that is the first to describe the use of HIFU in the treatment of BCC.

## 1. Introduction

Basal cell carcinoma (BCC) is the most prevalent form of skin cancer, particularly in the populations of Europe, Australia, and the USA [1]. This type of cancer, known for its slow growth and localized nature, has become a crucial concern in the field of dermatological oncology [2,3]. Notably, the incidence of BCC is anticipated to increase, correlated with the aging demographic trends in these regions [4]. This trend underscores the need for effective treatment strategies and highlights the burden BCC places on healthcare systems [5].

The traditional therapeutic arsenal for BCC primarily includes various surgical methods, such as excision, electrodesiccation and curettage (EDC), cryosurgery, and Mohs micrographic surgery [6]. These techniques, while effective, often face constraints, especially when treating facial lesions due to aesthetic and functional considerations [7]. Consequently, there is a growing interest in exploring and developing non-surgical alternatives that can offer equally effective outcomes with minimal aesthetic impact. These non-surgical options include destructive techniques (like curettage alone, cryosurgery, or electrodesiccation), photodynamic therapy, topical drugs (such as 5-fluorouracil, imiquimod, ingenol mebutate), radiotherapy, and hedgehog pathway inhibitors [8,9,10,11,12,13,14,15]. When choosing between these options, considerations include the location, tumor size, histopathological subtype, recurrence, and patient preference [16].

High-Intensity Focused Ultrasound (HIFU) represents a novel approach in this direction. HIFU is a non-invasive therapeutic technique that leverages focused ultrasound waves to selectively target and ablate pathological tissues [17]. The precision of HIFU technology, allowing for adjustable depth and energy concentration, makes it a promising tool for addressing various dermatological conditions [18]. Its application ranges from cosmetic procedures to the treatment of more serious conditions like skin cancers [19]. HIFU’s ability to concentrate energy at specific depths enables the effective targeting of skin layers with minimal damage to the surrounding healthy tissues [20,21,22]. Clinical observations have highlighted the potential of HIFU in dermatology. For instance, its use in treating conditions such as actinic keratosis, hypertrophic scars, and certain types of benign skin tumors has been documented with encouraging outcomes [19,23]. The versatility of HIFU, in terms of its adjustable parameters, broadens its applicability across a range of dermatological conditions, offering a less invasive alternative to traditional surgical methods [24].

Our study marks a significant milestone in the use of HIFU technology, as we present the first-ever pilot study with the successful removal of 15 BCC lesions in eight patients using this innovative technique. The procedure effectively eradicated BCC lesions with only minimal scarring, underscoring HIFU’s potential as an important approach in the non-invasive treatment of skin cancer. The subsequent sections of this paper will detail the methodology, outcomes, and broader implications of our study, shedding light on the transformative role HIFU could play in the future of dermatological cancer treatment.

## 2. Materials and Methods

### 2.1. Study Population and Lesion Selection

The investigation encompassed a cohort of eight patients with previous BCC history, comprising seven females and one male, who presented with a collective sum of 15 histopathologically confirmed lesions attributed to basal cell carcinoma (BCC). The age distribution among the participants was diverse, with individual ages of 74, 40, 73, 74, 70, 51, 61, and 67, culminating in a mean age of 63.75 years. Each lesion was subjected to a rigorous dermoscopic evaluation and subsequently chosen for intervention based on distinct clinical and dermoscopic criteria indicative of BCC. The clinical trial (N CT05698706) is ultimately designed to bring together a larger group of patients, and the results presented here are among the first cases we have described. Based on European consensus-based interdisciplinary guidelines for the diagnosis and treatment of basal cell carcinoma, the majority of cases were classified as I—low risk common BCC, and there were only two locations classified as IIa—common BCC, but somewhat DTT (temple and nose [25]).

### 2.2. Dermoscopic Evaluation

The clinical evaluation of each lesion in the study was meticulously conducted using the Fotofinder Medicam 1000 device. Although the protocol of the clinical study did not include taking dermoscopic images at every visit, researchers opted to do so in order to more thoroughly visualize the healing process. Dermoscopic features taken into consideration included a variety of vascular patterns such as linear branching, short linear, looped, bent, and glomerular vessels; segmental and radial lines; angular lines; and pigment structures like small and large gray-blue globules, hyperpigmented microcircles, white lines, and small non-structured white areas. Additionally, non-structured pink areas, white-yellow globules, and yellow globules representing ulceration were also assessed.

### 2.3. High-Intensity Focused Ultrasound (HIFU) Treatment

System ONE-M (TOOsonix A/S, Hoersholm, Denmark), operating at a frequency of 20 MHz, was employed for HIFU therapy. Ultrasound scanning using the Dermascan^®^C system (Cortex Technology ApS, Aalborg, Denmark) was conducted prior to the treatment, facilitating the accurate measurement of the depths of skin lesions. This step was crucial for selecting the appropriate HIFU handpiece tailored to the focal penetration depth required. In preparation for the procedure, the transducer chamber of the device was filled with deionized (DI) water and sealed with polyethylene film. Acoustic coupling was achieved using Parker Aquasonic 100^®^ ultrasound gel (Parker Laboratories Inc., Fairfield, NJ, USA). HIFU treatment parameters were individually customized to each type of skin lesion. The TOOsonix ONE-M offered four handpieces with focal penetration depths of 0.8, 1.3, 1.8, and 2.3 mm. The energy delivered by HIFU ranged from 0.7 to 1.3 J per exposure, with a duration of 150 ms for each dose. Treatments were carried out in a contiguous fashion, ensuring a 1 to 2 mm separation between doses for comprehensive lesion coverage, while maintaining a minimal circumferential margin of approximately 1 mm. The interval between subsequent exposures was maintained at around 1–2 s. Real-time monitoring of the treatment’s progress and status was consistently performed using the integrated dermoscopic imaging system. Notably, based on previous experience, pre-treatment topical anesthesia or local intralesional injections were not employed. Table 1 enumerates individual patient data, detailing the specific HIFU procedure parameters including transducer depth, pulse duration, energy per pulse, number of pulses administered, and the overall treatment time for each patient.

### 2.4. Post-Treatment Observations

Following the administration of High-Intensity Focused Ultrasound (HIFU) treatment, a detailed and systematic approach to post-treatment observation was employed. The protocol initiated with an evaluation for clinical signs of recurrence in the treated areas, focusing on identifying any indications of the condition’s reappearance. This aspect was critical in assessing the long-term efficacy of the HIFU therapy. Concurrently, a comprehensive categorization of expected adverse reactions during the healing period was conducted. This involved utilizing a graded scale, ranging from 0 for no reaction or a normal state to 3 for severe reactions, to document various reactions such as transient urticaria, edema, erythema, bloody purpura, inflammation, crust formation, hyperpigmentation, hypopigmentation, and scarring. This evaluation was instrumental in understanding the side effects and the healing trajectory post-treatment. Incorporating the patient perspective, the protocol also included a patient-based evaluation of the healing process. Patients were asked to rate their satisfaction with the healing process compared to their initial expectations, providing insights into the subjective experience of recovery. Moreover, the protocol encompassed a quantitative assessment of pain and itching experienced in the treated areas post-HIFU. Patients rated their discomfort on a scale from 0, indicating no pain or itching, to 10, signifying the worst possible pain or most intense itching. This assessment allowed for a nuanced understanding of the patient’s comfort and the treatment’s tolerability.

Overall, this structured and multi-dimensional post-treatment observation protocol offered a comprehensive perspective on both the clinical outcomes and patient experiences following HIFU therapy.

### 2.5. Ethical Compliance

Informed consent was obtained from all participants, and the study adhered to the ethical principles outlined in the Helsinki Declaration II. Approval for the study was granted by the Bioethics Committee of the Lower Silesian Chamber of Physicians in Wroclaw, with the assigned number 05/BOBD/2022.

## 3. Results

### 3.1. Clinical and Dermoscopic Involution of HIFU-Treated Basal Cell Carcinoma

The application of High-Intensity Focused Ultrasound (HIFU) in the treatment of basal cell carcinoma (BCC) induces immediate tissue responses, which are observable both clinically and dermoscopically. Following HIFU treatment, there is an immediate whitening of the treated area, known as blanching, which is indicative of protein denaturation and coagulative necrosis due to the thermal effects of the focused ultrasound. Accompanying this whitening is edema, commonly referred to as swelling, which represents a localized inflammatory response to the thermal insult. When employing HIFU transducers with focal depths greater than 0.8 mm, a more sustained response is noted, with a crust formation persisting beyond two weeks. This is contrasted by the use of 0.8 mm focal depth transducers, where crust formation typically resolves within a two-week period. In instances where a 0.8 mm transducer is utilized, the post-HIFU landscape is often characterized by a transient crust, which, upon resolution, reveals numerous healing processes. Dermoscopically, this post-crust phase is marked by an array of vascular changes, including fine arborizing vessels, radially aligned vessels, and looped linear vessels, all of which reflect the neovascularization phase of wound healing. The treated area may also exhibit varying degrees of pinkish hue, which is proportional to the intensity of the healing inflammatory process. Concurrently, a multitude of white structures becomes evident, particularly when examined under polarized dermoscopy. These structures, which may manifest as white lines or broader white areas, are representative of fibrotic changes within the dermal matrix. The fibrosis, a byproduct of the wound healing cascade, underscores the reparative processes following the HIFU-induced tissue injury. Three months following High-Intensity Focused Ultrasound (HIFU) treatment for basal cell carcinoma (BCC), the dermatoscopic images reveal signs indicative of the healing process. Fibrosis is evident, characterized by changes in tissue texture and the appearance of reticular linear vessels, which are suggestive of new collagen formation and revascularization, respectively. By the six-month mark, the lesion demonstrates a notable reduction in pigmentation, with the color becoming paler than the surrounding skin. This hypopigmentation is accompanied by the presence of singular white lines, a dermoscopic sign of fibrotic strands, which typically indicates ongoing remodeling of the extracellular matrix within the dermis. Over time, these fibrotic changes may diminish and possibly disappear by the one-year follow-up. The residual features at the six-month interval may include monomorphic reticular vessels, which are linear and arranged in a net-like pattern. The persistence of these vessels, while associated with the healing process, is also a feature commonly seen in benign dermal conditions. Their uniform appearance is generally not indicative of malignancy and can be considered a reassuring sign of the benign nature of the healing process.

The Figure 1, Figure 2, Figure 3, Figure 4, Figure 5, Figure 6, Figure 7, Figure 8, Figure 9, Figure 10, Figure 11, Figure 12, Figure 13, Figure 14, Figure 15, Figure 16, Figure 17, Figure 18, Figure 19, Figure 20, Figure 21, Figure 22, Figure 23, Figure 24, Figure 25, Figure 26, Figure 27 and Figure 28 alternately display ultrasound images and dermoscopic observations of the treated basal cell carcinoma (BCC) lesions. These images provide a visual representation of the textural and vascular changes observed during different stages of the healing process post-HIFU treatment, demonstrating the involution from immediate tissue responses to the long-term healing and remodeling of the skin.

Dermoscopic evaluation of treated lesions is summarized in Table 2, Table 3, Table 4 and Table 5.

### 3.2. Adverse Event Incidence Post-HIFU Treatment: Symptomatology and Frequency Analysis

Table 6 provides a detailed analysis of symptom occurrence percentages in patients following High-Intensity Focused Ultrasound (HIFU) treatment, distinctly categorized into various post-treatment intervals to elucidate the temporal progression of clinical manifestations. In the immediate aftermath of the treatment, spanning from 2 to 10 min, the most prevalent symptoms were edema, experienced by a majority of the patients (*n* = 14, 93.3%), and erythema (*n* = 12, 80%). This phase also witnessed notable instances of transient urticaria (*n* = 10, 66.7%) and hemorrhagic purpura (*n* = 3, 20%), reflecting the immediate response of the tissue to the HIFU therapy.

At the two-week post-treatment mark, a significant shift in the symptom profile was observed. Crust formation became the most prominent symptom (*n* = 7, 46.7%), indicating the onset of the healing process, while the continued presence of erythema (*n* = 4, 26.7%) suggested ongoing inflammatory reactions. Additionally, cases of hemorrhagic purpura and inflammation were recorded (*n* = 1 each, 6.7%), along with a slight increase in instances of inflammation (*n* = 2, 13.3%), underscoring the varied skin responses at this stage.

Progressing to the three-month evaluation, the data revealed the persistence of erythema in a subset of patients (*n* = 6, 40%), pointing to prolonged inflammatory activity. A notable increase in scarring (*n* = 5, 33.3%) was also observed, indicative of the tissue remodeling phase. Additionally, individual instances of hemorrhagic purpura and hyperpigmentation were reported (*n* = 1 each, 6.7%), highlighting the diverse dermatological impacts of the treatment.

At the six-month assessment, an escalation in scarring (*n* = 6, 40%) was evident, suggesting long-term effects of tissue remodeling. The emergence of hyperpigmentation and hypopigmentation (*n* = 2 each, 13.3%) at this stage brought to light the potential for persistent pigmentary changes. Moreover, the continued presence of erythema in a few patients (*n* = 2, 13.3%) at this late stage suggested sustained inflammatory processes or delayed healing in certain cases.

### 3.3. Quantitative Evaluation of Post-HIFU Treatment Patient Responses: A Longitudinal Assessment

#### 3.3.1. Immediate Post-HIFU Treatment Observations

In the immediate post-procedure phase of HIFU, a preponderant majority (87.5%, *n* = 7) exhibited only mild adverse effects, indicated by a score of 1. Contrastingly, a smaller subset (12.5%, *n* = 1) reported experiencing moderate adverse effects, evidenced by a score of 2. Pain levels during the procedure varied across the cohort: 37.5% (*n* = 3) reported a moderate pain intensity of 3 on a 0–10 scale, while 25% (*n* = 2) experienced divergent pain levels, one with a minimal intensity of 1 and another with a more substantial intensity of 7. Shortly after post-treatment, a significant proportion of the cohort (87.5%, *n* = 7) reported no pain, scoring 0, while a minority (12.5%, *n* = 1) experienced slight pain, with scores ranging from 1 to 2. All participants (100%, *n* = 8) reported the absence of itching post-procedure, a uniform score of 0 across the board. Regarding comparative methods, surgical excision was reported by 62.5% (*n* = 5) of patients, while the remaining 37.5% (*n* = 3) underwent cryotherapy. Other adverse effects were notably infrequent, with 75% (*n* = 6) of patients reporting none, albeit specific symptoms like significant palpation pain and prolonged sensory disturbances were observed. Overall, the intensity of adverse effects was predominantly mild, with 62.5% (*n* = 5) scoring 1, and moderate in 37.5% (*n* = 3), scoring 2.

#### 3.3.2. Two Weeks following HIFU Treatment

Two weeks after treatment, all participants (100%, *n* = 8) continued to report mild adverse effects, consistently scoring 1. A decrease in pain sensation was noted, with 75% (*n* = 6) reporting no pain (score 0), and 25% (*n* = 2) experiencing mild pain, with scores of 1 and 3. Itching was absent in a majority (75%, *n* = 6), scoring 0, and mild itching was noted by 25% (*n* = 2), scoring 1 and 2. The absence of other adverse effects was unanimous among all patients (100%, *n* = 8) at this interval. The assessment of adverse effect intensity uniformly yielded mild effects for the entire cohort, scoring 1. These observations applied to the entire period since the HIFU treatment.

#### 3.3.3. Three Months Post-HIFU Procedure

Three months after treatment, the pattern of adverse effects showed 87.5% (*n* = 7) with mild effects, and a notable shift as 12.5% (*n* = 1) reported no adverse effects, scoring 0. A marked improvement in pain levels was observed, with all patients (100%, *n* = 8) indicating no pain, unanimously scoring 0. The majority (75%, *n* = 6) continued to report no itching, scoring 0, while a minority (25%, *n* = 2) still experienced mild itching, scoring 1. Other adverse effects were minimal, with 87.5% (*n* = 7) reporting no effects, though isolated incidents of paresthesia, punctate pain at the treatment site, and lesion fibrosis were recorded. The intensity of adverse effects was largely mild for 62.5% (*n* = 5), moderate for 25% (*n* = 2), and non-existent for 12.5% (*n* = 1). These observations applied to the entire period since the HIFU treatment.

#### 3.3.4. Six Months after HIFU Treatment

Six months after treatment, the patient-reported outcomes indicated stability, with 75% (*n* = 6) experiencing mild adverse effects, 12.5% (*n* = 1) moderate effects, and 12.5% (*n* = 1) reporting no effects. An enduring absence of pain was noted among all patients (100%, *n* = 8), consistently scoring 0. Itching was reported as non-existent across the board (100%, *n* = 8), signifying a return to the initial post-treatment state. There were no reports of other adverse effects from the entire cohort (100%, *n* = 8) at the six-month follow-up. The total intensity of adverse effects revealed a majority experiencing mild effects (75%, *n* = 6), with a smaller fraction encountering moderate (12.5%, *n* = 1) and no effects (12.5%, *n* = 1). To provide a clear and systematic overview of patient outcomes post-HIFU treatment, Table 7 illustrates the progression of responses at four key intervals: immediately post-treatment, two weeks, three months, and six months. These observations applied to the entire period since the HIFU treatment.

## 4. Discussion

Our study of the utility of high-intensity focused ultrasound (HIFU) in the treatment of basal cell carcinoma (BCC) offers new possibilities in the treatment of dermatological cancers. Basal Cell Carcinoma, as the predominant form of non-melanoma skin cancer, represents a significant clinical challenge, particularly in its predilection for facial involvement [26]. Approximately 80% of all BCC cases manifest on the face, with the nose accounting for 25% to 30% of these occurrences [27]. Notably, the ear/nose region carries a relative risk of incomplete excision of BCC that is 2.5 times higher compared to other anatomical regions of the body [28]. The anatomical complexity and cosmetic significance of the facial region, especially the nose, which harbors a substantial proportion of BCC cases, necessitate treatment modalities that balance oncologic efficacy with aesthetic preservation [29].

HIFU is a new treatment modality that has demonstrated its high efficacy in the treatment of many premalignant lesions and skin cancers over the past few years [19,30]. In this context, our study’s exploration of HIFU emerges as a significant contribution. HIFU, with its non-invasive nature and precision in dosing of the therapeutic energy to the diseased tissue, stands as an innovative alternative to traditional surgery. It circumvents the common drawbacks of surgical interventions, particularly scarring and extensive tissue damage. The ability of HIFU to customize depth and energy deposition allows for precise dosing of the therapeutic energy of pathological tissue, thus preserving adjacent healthy structures [19]. Our study marks a significant advancement in dermatological oncology, focusing on the application of HIFU for the treatment of BCC. We successfully treated 15 BCC lesions in eight patients, showcasing HIFU’s efficacy and minimal scarring. To our knowledge, this is the first clinical study of its scale on HIFU for BCC, as previous instances primarily consisted of case studies; after a six-month observation period, we observed no recurrences of the treated BCC lesions, underscoring the efficacy of HIFU. Building on these clinical outcomes, dermoscopic examination provided further insights into the healing process post-HIFU treatment. The involution of BCC treated with HIFU demonstrates distinct phases of skin healing. Initial blanching and edema transitioned to crust formation, followed by neovascularization, characterized by fine arborizing, radially aligned, and looped linear vessels—key indicators of reparative processes. Over six months, these vascular changes evolved into fibrotic signs like white lines and reticular vessels, signifying collagen remodeling and benign healing. These findings not only underscore HIFU’s effectiveness in non-invasive BCC treatment but also highlight its potential role in supporting tissue repair and regeneration.

Importantly, our study revealed that during the healing process, patients mostly identified the HIFU method as inducing less discomfort compared to other treatments. This finding is particularly significant, considering that treatments like Photodynamic Therapy (PDT), a common alternative for non-melanoma skin cancers, are often associated with notable pain during illumination [31,32,33,34]. Further reinforcing the patient-centered benefits of HIFU, the six-month follow-up data revealed that a significant majority of the cohort, 75%, reported being ‘Very Satisfied’ with the treatment outcomes, while the remaining 25% were categorized as ‘Satisfied’. This distribution of satisfaction levels reflects strong acceptance of HIFU among patients as a preferred treatment option for BCC. Moreover, the cost-effectiveness of HIFU, in comparison to surgical methods, adds another dimension to its clinical utility. While surgical excision remains the gold standard, it often leads to significant scarring and higher costs due to the need for reconstructive procedures [35,36]. The HIFU method requires the purchase of the device. There is no need for nurse assistance, sutures, or anesthesia. Although the probe does wear out, each shot costs approximately $0.25 when calculating the cost of the probe.

Our study has shown promising results in treating BCC with HIFU, emphasizing its potential as a non-invasive alternative to traditional surgery. However, incorporating our recommendations into this discussion enhances our narrative by providing detailed guidelines for optimizing HIFU treatment efficacy and safety. We advocate for the use of probes with penetration depths exceeding the tumor infiltration depth identified via pre-procedure ultrasonography by at least 0.2 mm. This precision in probe selection, based on infiltration depth, ensures targeted ablation of pathological tissues while preserving surrounding healthy structures. For instance, for tumors with infiltration up to 0.6 mm, a 0.8 mm probe is recommended, and so forth, up to a 2.3 mm probe for infiltrations up to 2 mm. Furthermore, our observations of the tissue response to HIFU pulses, evidenced by the appearance of white circles of varying diameters depending on the treated tissue type, underscore the importance of overlapping the radii of these circles by at least 0.1 mm to encompass the entire treatment area. This technique ensures comprehensive coverage and treatment efficacy. Additionally, in cases of basal cell carcinoma with infiltration greater than 1.6 mm, employing a dual-probe strategy—initially using a deeper probe followed by a shallower one—optimizes the treatment by leveraging tissue permeability to ultrasound waves for effective ablation. Our study also highlights the importance of avoiding multiple pulse depositions in the same spot if an ablative effect is visible, to prevent tissue damage and bleeding. We recommend a minimum interval of 7 days from biopsy to the HIFU procedure to allow for healing, thus not affecting the treatment outcome negatively.

However, it is important to note that one of the patients’ lesions was located on the nasal area. The nose is a favored site for perineural invasion (PNI) in BCC [37,38]. Perineural invasion in BCC is an important pathological finding in which tumor cells invade the spaces surrounding nerves. PNI indicates more aggressive disease and is associated with higher recurrence rates and potentially worse outcomes. PNI can be incidental (microscopic without clinical signs) or clinical (with symptoms such as pain or nerve dysfunction). Studies show that the incidence of PNI in BCC varies from 0.178% to 10%, depending on the method of detection. Mohs micrographic surgery (MMS), which uses frozen scrapings to examine margins, tends to detect PNI more often than conventional pathology because of its detailed examination of horizontal tissue scrapings. BCCs with PNI, especially in the nasal region, tend to be more aggressive and have a higher propensity for recurrence. This is due to the dense neural network in the nasal area, which provides more pathways for the tumor to spread. Detecting PNI in the nasal region can be challenging. The complex anatomy of the nasal region and the high density of nerves mean that even small, seemingly localized BCCs may have already invaded the perinuclear spaces, making careful histopathological examination crucial.

The adoption of dermoscopy for precise contouring of the treatment area, alongside the recommendation of specific ultrasound exposure times and energy ranges, demonstrates our dedication to enhancing High-Intensity Focused Ultrasound (HIFU) treatment protocols for Basal Cell Carcinoma (BCC). These guidelines are designed to maximize therapeutic efficacy while minimizing adverse effects, in line with our findings on HIFU’s effectiveness, minimal scarring, and high patient satisfaction. The integration of these recommendations emphasizes HIFU’s clinical value and its contribution to advancing dermatologic oncology towards less invasive, aesthetically pleasing, and patient-preferred methods. Looking ahead, it is imperative to assess the long-term efficacy of these refined HIFU protocols, their performance compared to other treatments, and their effectiveness across different BCC subtypes, including nodular BCC. This future research will be crucial in solidifying HIFU’s role in the skin cancer treatment spectrum, offering a compelling combination of therapeutic success, cosmetic preservation, and patient contentment. This study has several limitations such as the possibility of selection bias, small sample size and short observation time. However, this is a pilot study, and the purpose of this study was to pre-report preliminary data.

## 5. Conclusions

In this pioneering investigation, we evaluated the efficacy and safety of High-Intensity Focused Ultrasound (HIFU) therapy for the treatment of Basal Cell Carcinoma (BCC), revealing its potential as a novel, non-invasive treatment modality. Our results demonstrate a high success rate in the complete ablation of BCC lesions without any recurrences observed during the follow-up period, underscoring the effectiveness of HIFU as a viable alternative to traditional therapies. The safety profile of HIFU therapy was favorable, with minimal adverse events reported, highlighting its suitability for clinical application. Notably, HIFU therapy achieved superior cosmetic outcomes with minimal scarring, significantly enhancing patient satisfaction compared to conventional treatments. This minimal scarring and high patient satisfaction suggest a potential shift in the treatment paradigm for BCC, especially for lesions in cosmetically sensitive areas. The findings of this study advocate for the integration of HIFU into the therapeutic arsenal against BCC, suggesting a shift towards less invasive treatment strategies. Future research should focus on the long-term efficacy of HIFU, its utility across different BCC subtypes, and comparative studies with existing treatment modalities to fully establish its role in dermatological oncology. In summary, our investigation positions HIFU therapy as an important approach in the management of Basal Cell Carcinoma, promising a combination of clinical efficacy, safety, and patient-centered outcomes.

## Figures and Tables

**Figure 1:**
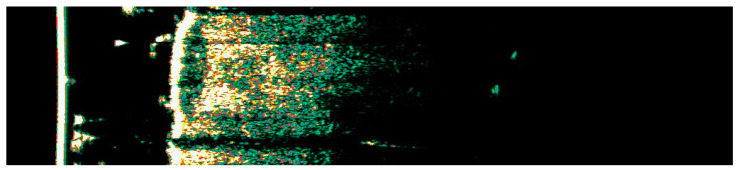
Ultrasonographic image of BCC on the arm.

**Figure 2:**
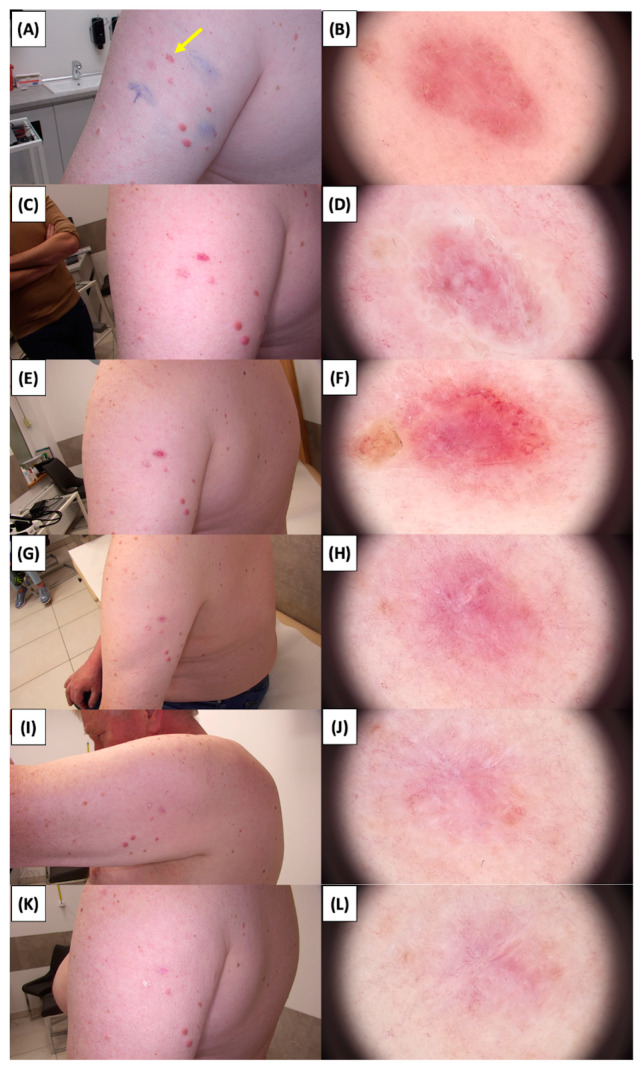
Involution of Basal Cell Carcinoma (BCC) on the Arm Treated with High-Intensity Focused Ultrasound (HIFU). (**A**) Macroscopic view before HIFU treatment; (**B**) Dermoscopic view before HIFU treatment; (**C**) Macroscopic view immediately after HIFU treatment; (**D**) Dermoscopic view immediately after HIFU treatment; (**E**) Macroscopic view 2 weeks post-HIFU; (**F**) Dermoscopic view 2 weeks post-HIFU; (**G**) Macroscopic view 3 months post-HIFU; (**H**) Dermoscopic view 3 months post-HIFU; (**I**) Macroscopic view 6 months post-HIFU; (**J**) Dermoscopic view 6 months post-HIFU; (**K**) Macroscopic view 1 year post-HIFU; (**L**) Dermoscopic view 1 year post-HIFU. Yellow arrows indicate the area of the treated lesion.

**Figure 3:**
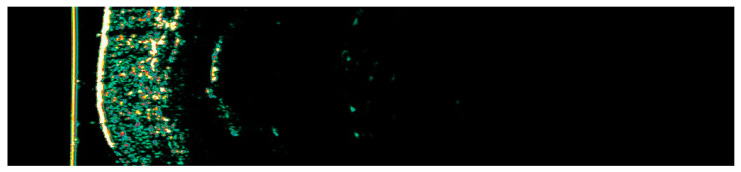
Ultrasonographic image of BCC on the cheek.

**Figure 4:**
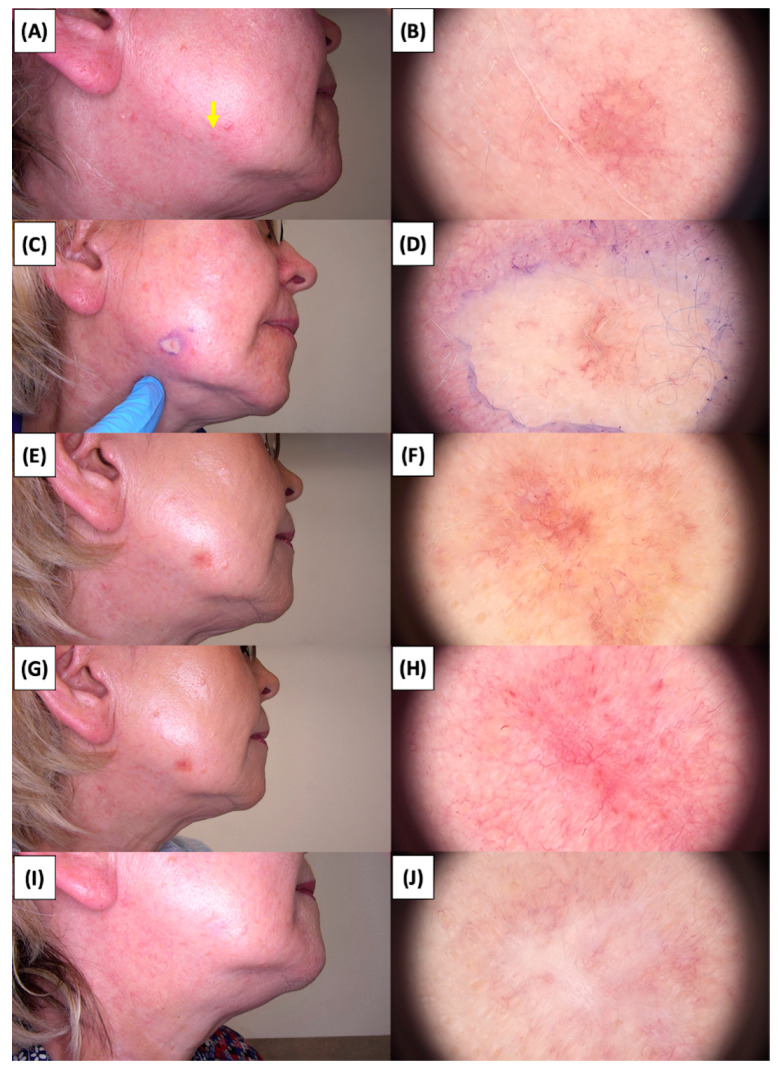
Involution of Basal Cell Carcinoma (BCC) on the cheek treated with High-Intensity Focused Ultrasound (HIFU). (**A**) Macroscopic view before HIFU treatment; (**B**) Dermoscopic view before HIFU treatment; (**C**) Macroscopic view immediately after HIFU treatment; (**D**) Dermoscopic view immediately after HIFU treatment; (**E**) Macroscopic view 2 weeks post-HIFU; (**F**) Dermoscopic view 2 weeks post-HIFU; (**G**) Macroscopic view 3 months post-HIFU; (**H**) Dermoscopic view 3 months post-HIFU; (**I**) Macroscopic view 6 months post-HIFU; (**J**) Dermoscopic view 6 months post-HIFU. The yellow arrow indicates the area of the lesion.

**Figure 5:**
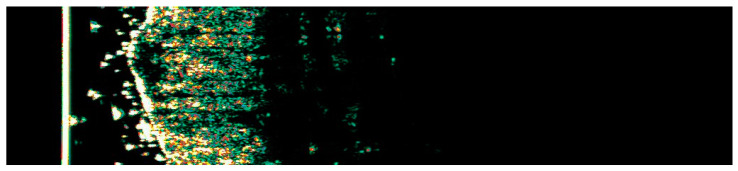
Ultrasonographic image of BCC on the temple.

**Figure 6:**
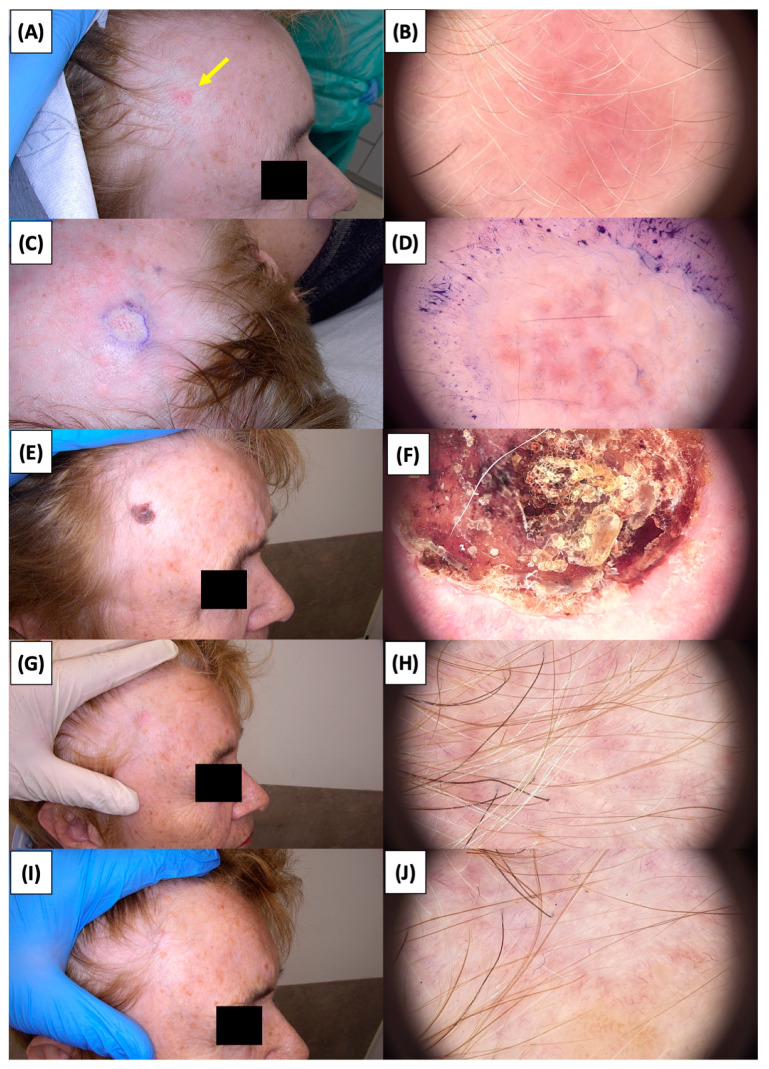
Involution of Basal Cell Carcinoma (BCC) on the temple treated with High-Intensity Focused Ultrasound (HIFU). (**A**) Macroscopic view before HIFU treatment; (**B**) Dermoscopic view before HIFU treatment; (**C**) Macroscopic view immediately after HIFU treatment; (**D**) Dermoscopic view immediately after HIFU treatment; (**E**) Macroscopic view 2 weeks post-HIFU; (**F**) Dermoscopic view 2 weeks post-HIFU; (**G**) Macroscopic view 3 months post-HIFU; (**H**) Dermoscopic view 3 months post-HIFU; (**I**) Macroscopic view 6 months post-HIFU; (**J**) Dermoscopic view 6 months post-HIFU. The yellow arrow indicates the area of the lesion.

**Figure 7:**
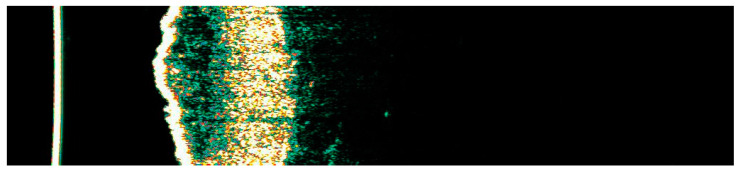
Ultrasonographic image of BCC on the nose.

**Figure 8:**
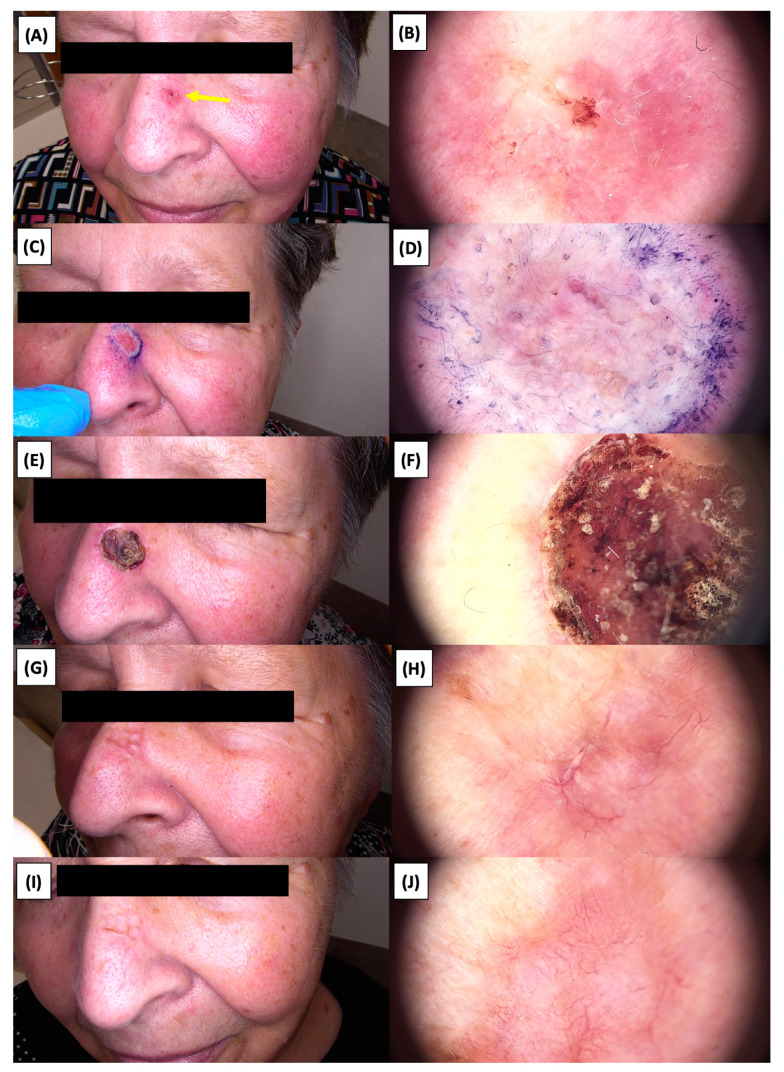
Involution of Basal Cell Carcinoma (BCC) on the nasal area treated with High-Intensity Focused Ultrasound (HIFU). (**A**) Macroscopic view before HIFU treatment; (**B**) Dermoscopic view before HIFU treatment; (**C**) Macroscopic view immediately after HIFU treatment; (**D**) Dermoscopic view immediately after HIFU treatment; (**E**) Macroscopic view 2 weeks post-HIFU; (**F**) Dermoscopic view 2 weeks post-HIFU; (**G**) Macroscopic view 3 months post-HIFU; (**H**) Dermoscopic view 3 months post-HIFU; (**I**) Macroscopic view 6 months post-HIFU; (**J**) Dermoscopic view 6 months post-HIFU. The yellow arrow indicates the area of the lesion.

**Figure 9:**
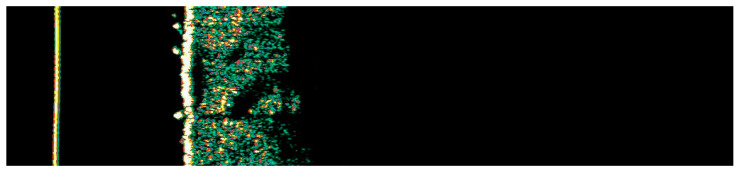
Ultrasonographic image of BCC on the thigh.

**Figure 10:**
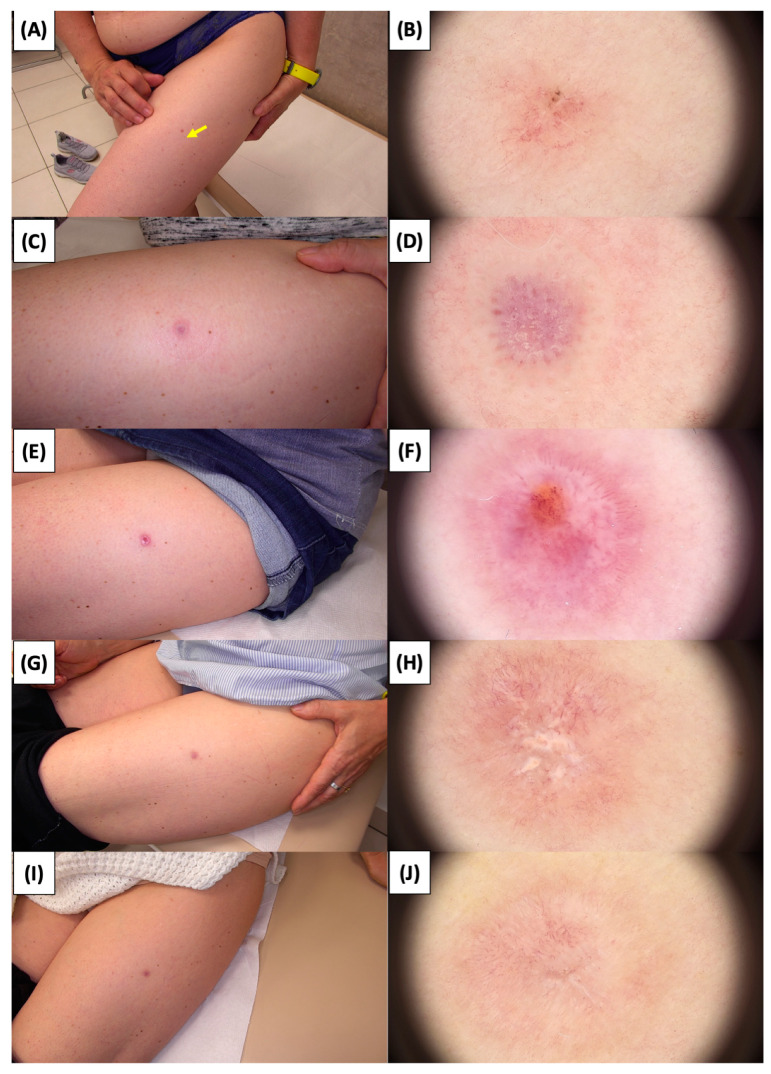
Involution of Basal Cell Carcinoma (BCC) on the thigh treated with High-Intensity Focused Ultrasound (HIFU). (**A**) Macroscopic view before HIFU treatment; (**B**) Dermoscopic view before HIFU treatment; (**C**) Macroscopic view immediately after HIFU treatment; (**D**) Dermoscopic view immediately after HIFU treatment; (**E**) Macroscopic view 2 weeks post-HIFU; (**F**) Dermoscopic view 2 weeks post-HIFU; (**G**) Macroscopic view 3 months post-HIFU; (**H**) Dermoscopic view 3 months post-HIFU; (**I**) Macroscopic view 6 months post-HIFU; (**J**) Dermoscopic view 6 months post-HIFU. The yellow arrow indicates the area of the lesion.

**Figure 11:**
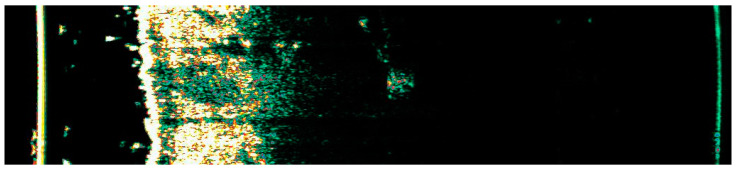
Ultrasonographic image of BCC on the upper arm.

**Figure 12:**
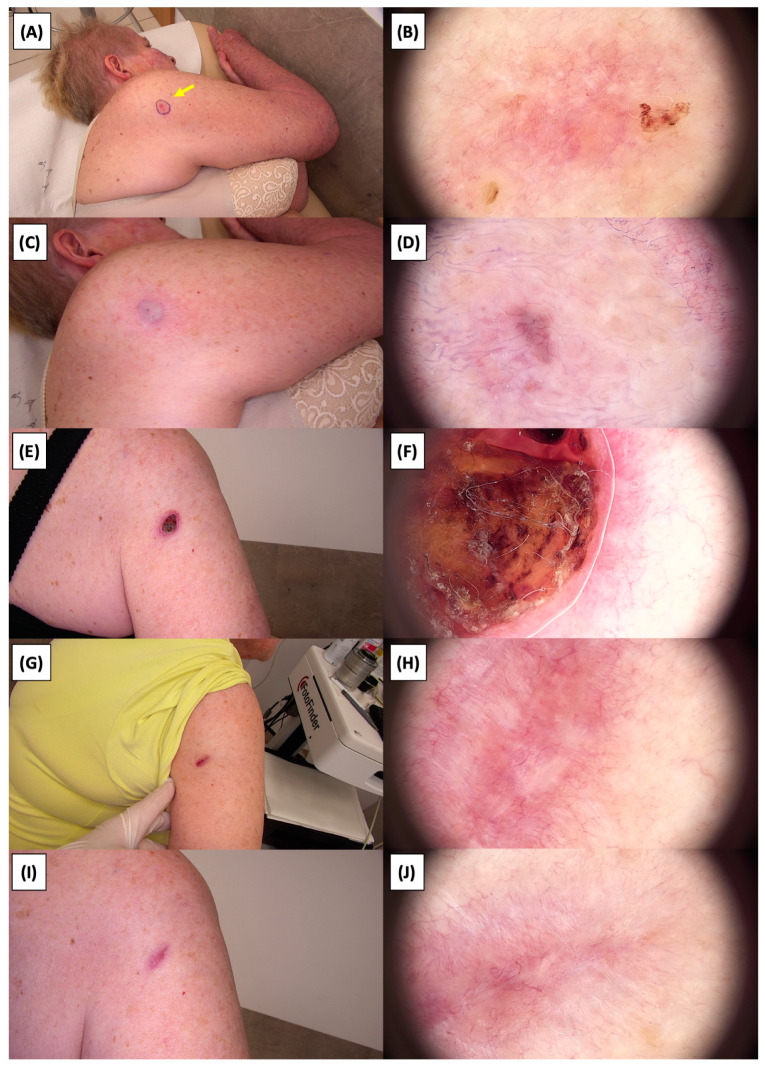
Involution of Basal Cell Carcinoma (BCC) on the upper arm treated with High-Intensity Focused Ultrasound (HIFU). (**A**) Macroscopic view before HIFU treatment; (**B**) Dermoscopic view before HIFU treatment; (**C**) Macroscopic view immediately after HIFU treatment; (**D**) Dermoscopic view immediately after HIFU treatment; (**E**) Macroscopic view 2 weeks post-HIFU; (**F**) Dermoscopic view 2 weeks post-HIFU; (**G**) Macroscopic view 3 months post-HIFU; (**H**) Dermoscopic view 3 months post-HIFU; (**I**) Macroscopic view 6 months post-HIFU; (**J**) Dermoscopic view 6 months post-HIFU. The yellow arrow indicates the area of the lesion.

**Figure 13:**
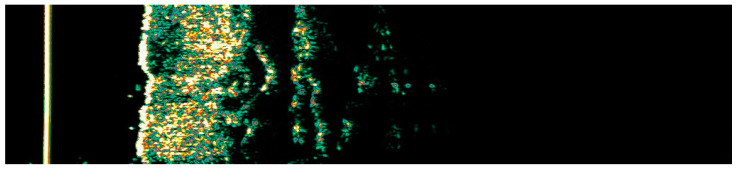
Ultrasonographic image of BCC on the neck.

**Figure 14:**
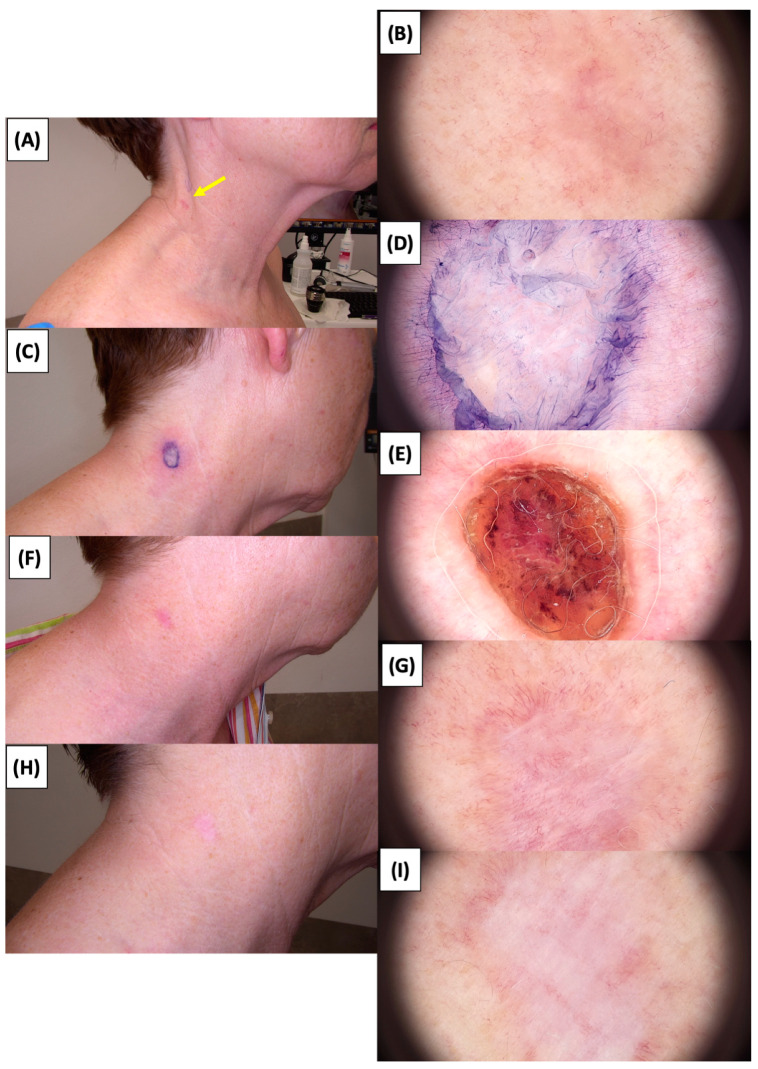
Involution of Basal Cell Carcinoma (BCC) on the neck treated with High-Intensity Focused Ultrasound (HIFU). (**A**) Macroscopic view before HIFU treatment; (**B**) Dermoscopic view before HIFU treatment; (**C**) Macroscopic view immediately after HIFU treatment; (**D**) Dermoscopic view immediately after HIFU treatment; (**E**) Macroscopic view 2 weeks post-HIFU; (**F**) Dermoscopic view 2 weeks post-HIFU; (**G**) Macroscopic view 3 months post-HIFU; (**H**) Dermoscopic view 3 months post-HIFU; (**I**) Macroscopic view 6 months post-HIFU. The yellow arrow indicates the area of the lesion.

**Figure 15:**
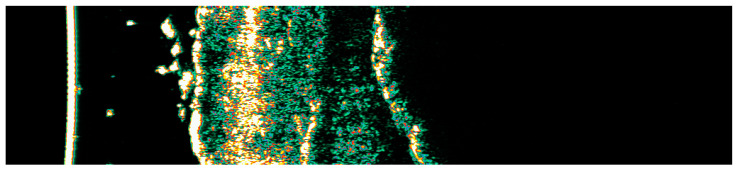
Ultrasonographic image of BCC on the temple.

**Figure 16:**
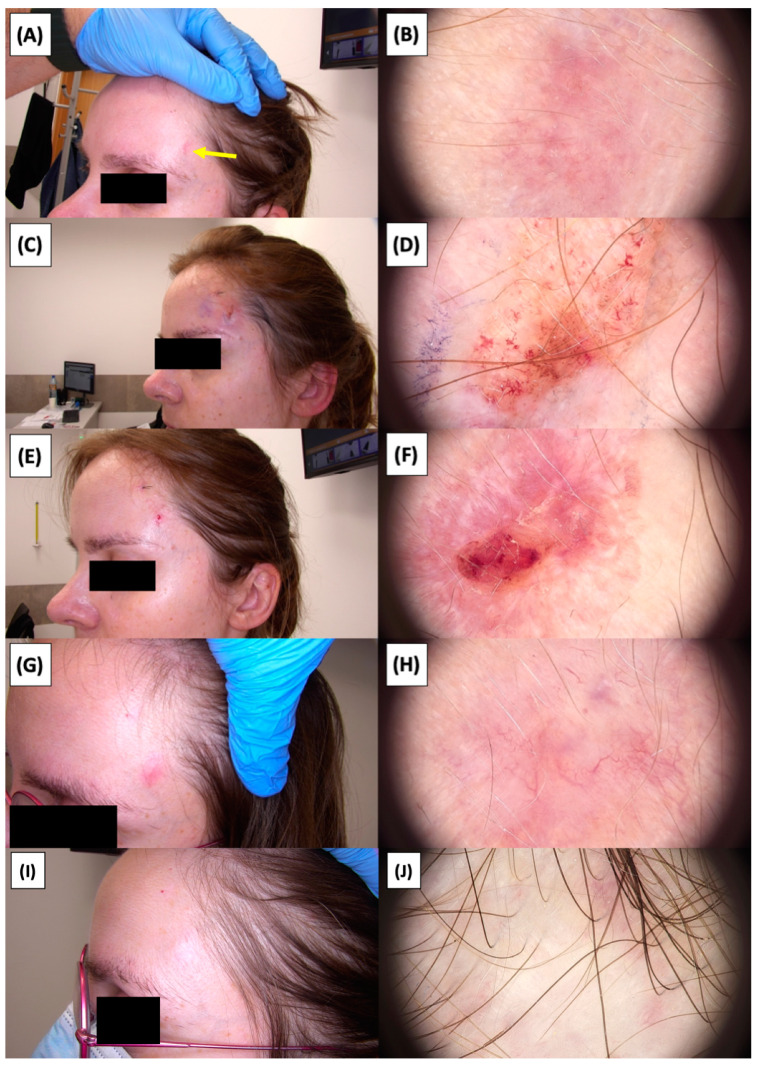
Involution of Basal Cell Carcinoma (BCC) on the temple treated with High-Intensity Focused Ultrasound (HIFU). (**A**) Macroscopic view before HIFU treatment; (**B**) Dermoscopic view before HIFU treatment; (**C**) Macroscopic view immediately after HIFU treatment; (**D**) Dermoscopic view immediately after HIFU treatment; (**E**) Macroscopic view 2 weeks post-HIFU; (**F**) Dermoscopic view 2 weeks post-HIFU; (**G**) Macroscopic view 3 months post-HIFU; (**H**) Dermoscopic view 3 months post-HIFU; (**I**) Macroscopic view 6 months post-HIFU; (**J**) Dermoscopic view 6 months post-HIFU. The yellow arrow indicates the area of the lesion.

**Figure 17:**
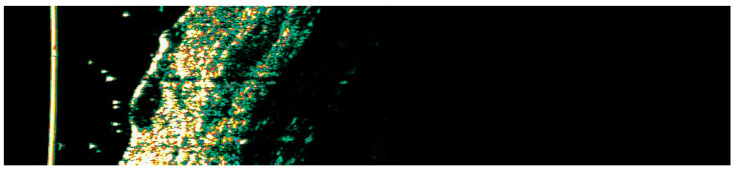
Ultrasonographic image of BCC on the chest.

**Figure 18:**
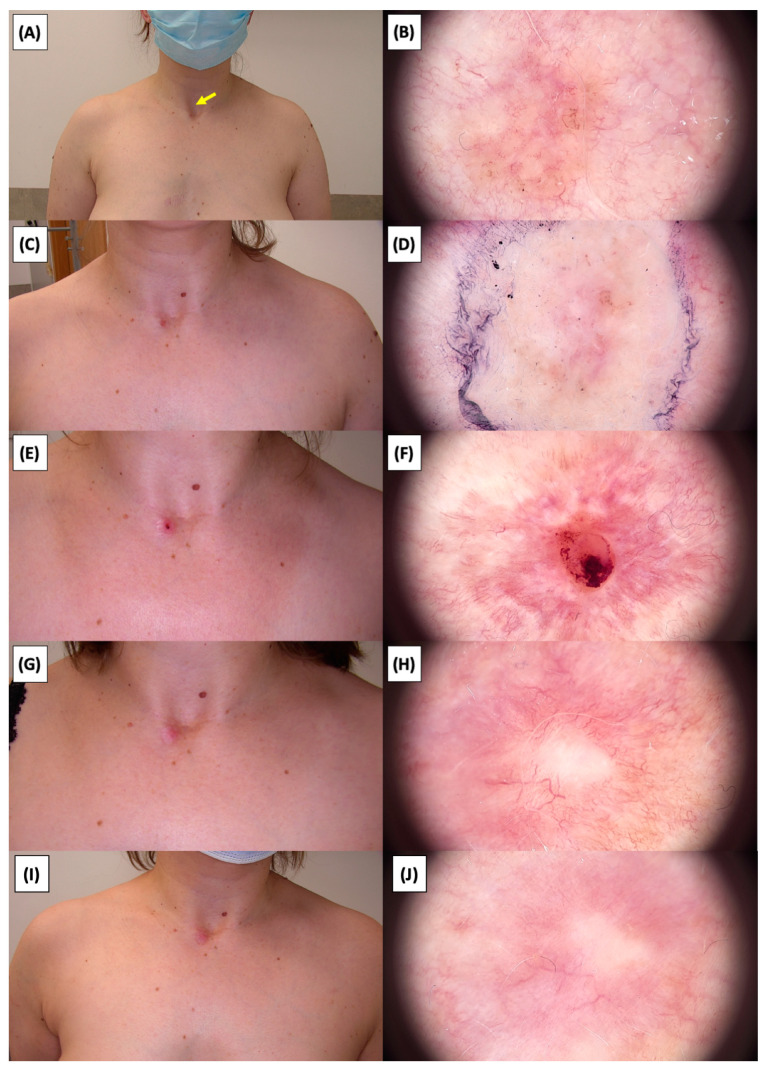
Involution of Basal Cell Carcinoma (BCC) on the chest treated with High-Intensity Focused Ultrasound (HIFU). (**A**) Macroscopic view before HIFU treatment; (**B**) Dermoscopic view before HIFU treatment; (**C**) Macroscopic view immediately after HIFU treatment; (**D**) Dermoscopic view immediately after HIFU treatment; (**E**) Macroscopic view 2 weeks post-HIFU; (**F**) Dermoscopic view 2 weeks post-HIFU; (**G**) Macroscopic view 3 months post-HIFU; (**H**) Dermoscopic view 3 months post-HIFU; (**I**) Macroscopic view 6 months post-HIFU; (**J**) Dermoscopic view 6 months post-HIFU. The yellow arrow indicates the area of the lesion.

**Figure 19:**
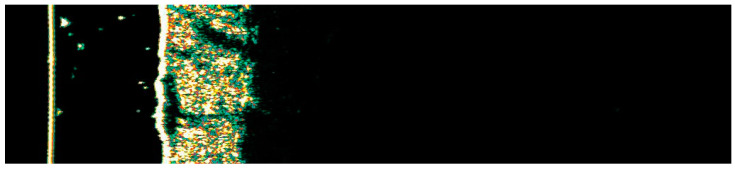
Ultrasonographic image of BCC on the arm.

**Figure 20:**
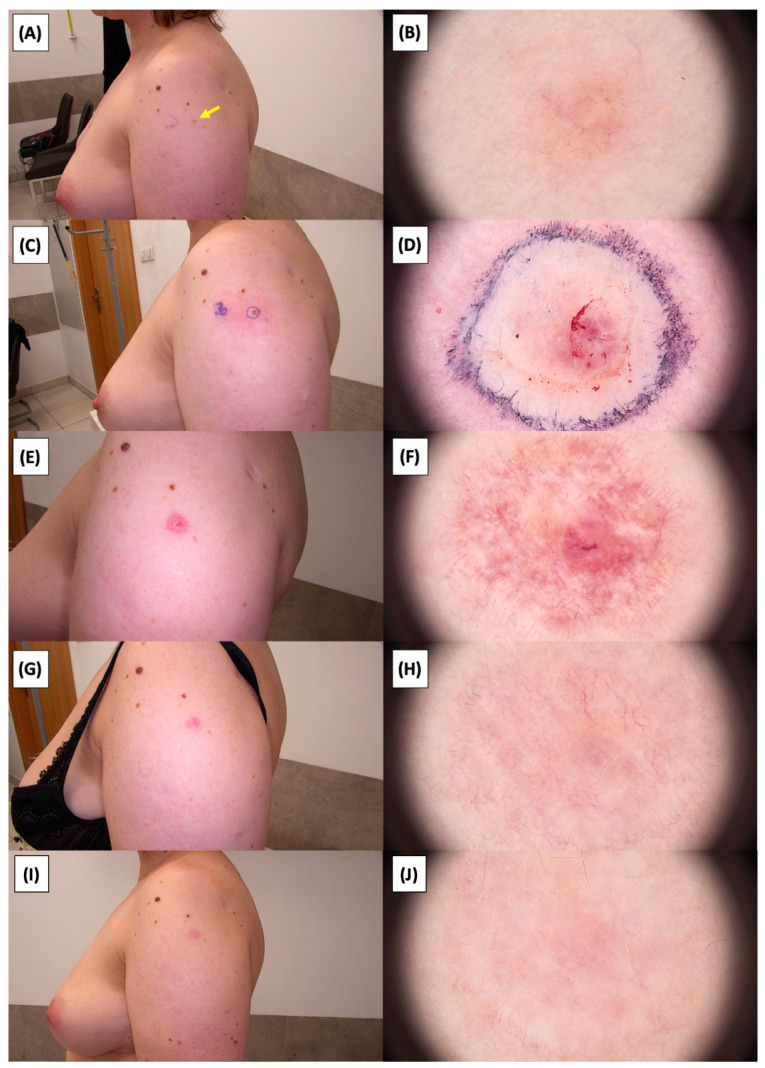
Involution of Basal Cell Carcinoma (BCC) on the arm treated with High-Intensity Focused Ultrasound (HIFU). (**A**) Macroscopic view before HIFU treatment; (**B**) Dermoscopic view before HIFU treatment; (**C**) Macroscopic view immediately after HIFU treatment; (**D**) Dermoscopic view immediately after HIFU treatment; (**E**) Macroscopic view 2 weeks post-HIFU; (**F**) Dermoscopic view 2 weeks post-HIFU; (**G**) Macroscopic view 3 months post-HIFU; (**H**) Dermoscopic view 3 months post-HIFU; (**I**) Macroscopic view 6 months post-HIFU; (**J**) Dermoscopic view 6 months post-HIFU. The yellow arrow indicates the area of the lesion.

**Figure 21:**
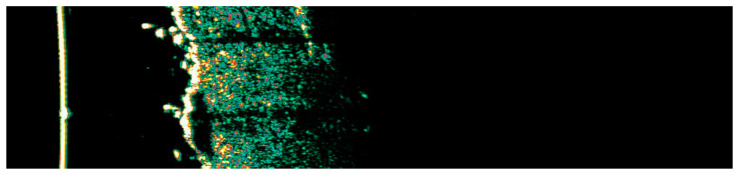
Ultrasonographic image of BCC on the back.

**Figure 22:**
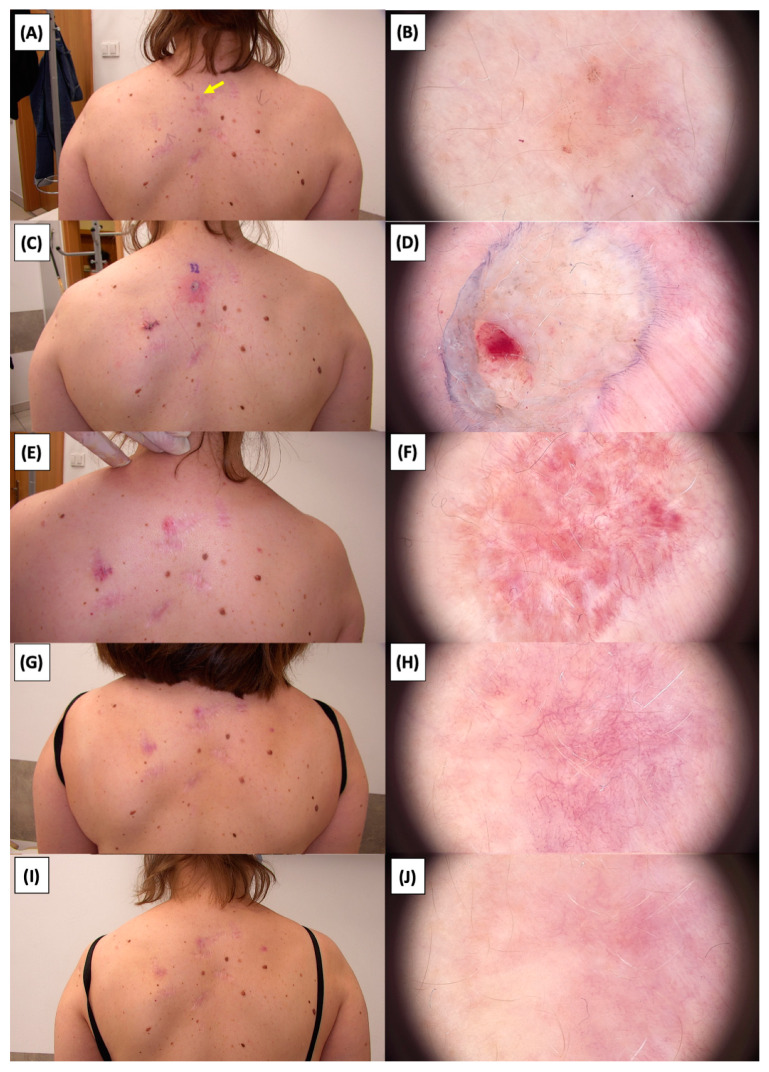
Involution of Basal Cell Carcinoma (BCC) on the back treated with High-Intensity Focused Ultrasound (HIFU). (**A**) Macroscopic view before HIFU treatment; (**B**) Dermoscopic view before HIFU treatment; (**C**) Macroscopic view immediately after HIFU treatment; (**D**) Dermoscopic view immediately after HIFU treatment; (**E**) Macroscopic view 2 weeks post-HIFU; (**F**) Dermoscopic view 2 weeks post-HIFU; (**G**) Macroscopic view 3 months post-HIFU; (**H**) Dermoscopic view 3 months post-HIFU; (**I**) Macroscopic view 6 months post-HIFU; (**J**) Dermoscopic view 6 months post-HIFU. The yellow arrow indicates the area of the lesion.

**Figure 23:**
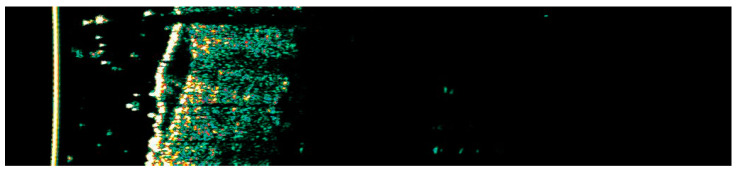
Ultrasonographic image of BCC on the back.

**Figure 24:**
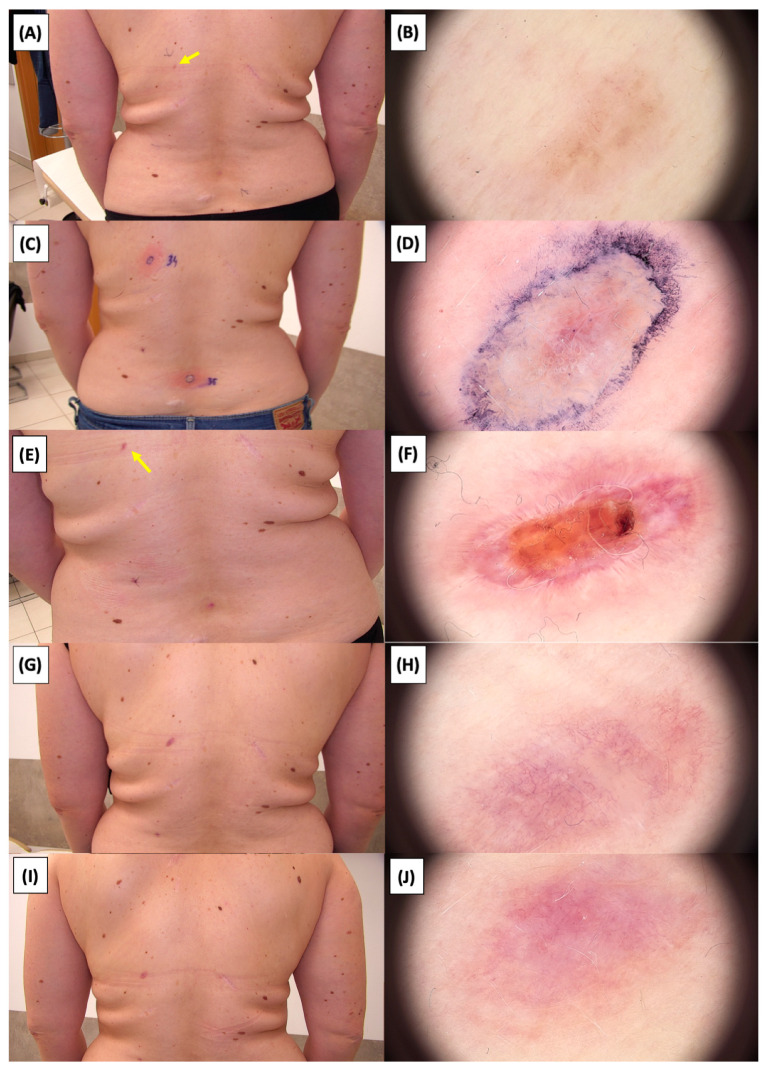
Involution of Basal Cell Carcinoma (BCC) on the back treated with High-Intensity Focused Ultrasound (HIFU). (**A**) Macroscopic view before HIFU treatment; (**B**) Dermoscopic view before HIFU treatment; (**C**) Macroscopic view immediately after HIFU treatment; (**D**) Dermoscopic view immediately after HIFU treatment; (**E**) Macroscopic view 2 weeks post-HIFU; (**F**) Dermoscopic view 2 weeks post-HIFU; (**G**) Macroscopic view 3 months post-HIFU; (**H**) Dermoscopic view 3 months post-HIFU; (**I**) Macroscopic view 6 months post-HIFU; (**J**) Dermoscopic view 6 months post-HIFU. The yellow arrow indicates the area of the lesion.

**Figure 25:**
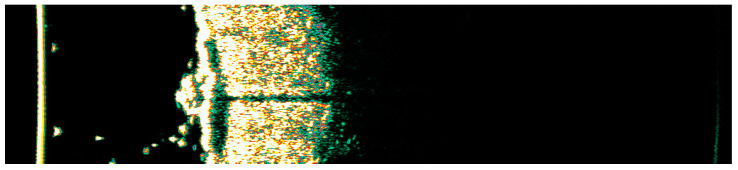
Ultrasonographic image of BCC on the lower back.

**Figure 26:**
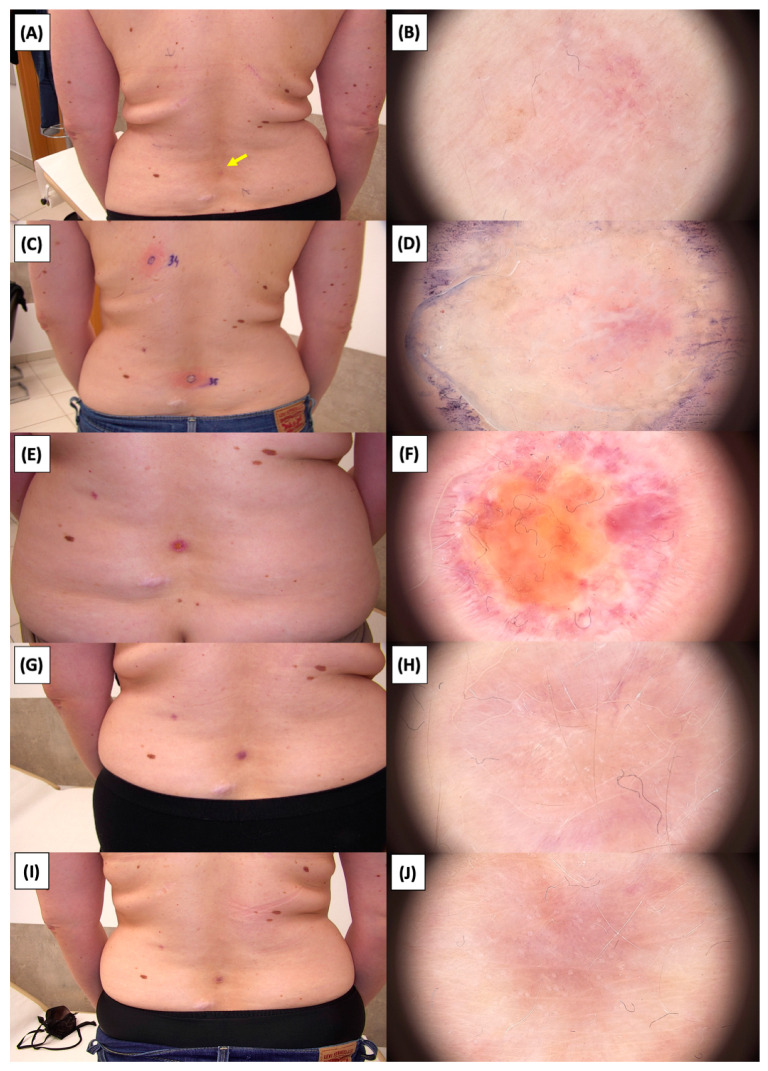
Involution of Basal Cell Carcinoma (BCC) on the lower back treated with High-Intensity Focused Ultrasound (HIFU). (**A**) Macroscopic view before HIFU treatment; (**B**) Dermoscopic view before HIFU treatment; (**C**) Macroscopic view immediately after HIFU treatment; (**D**) Dermoscopic view immediately after HIFU treatment; (**E**) Macroscopic view 2 weeks post-HIFU; (**F**) Dermoscopic view 2 weeks post-HIFU; (**G**) Macroscopic view 3 months post-HIFU; (**H**) Dermoscopic view 3 months post-HIFU; (**I**) Macroscopic view 6 months post-HIFU; (**J**) Dermoscopic view 6 months post-HIFU. The yellow arrow indicates the area of the lesion.

**Figure 27:**
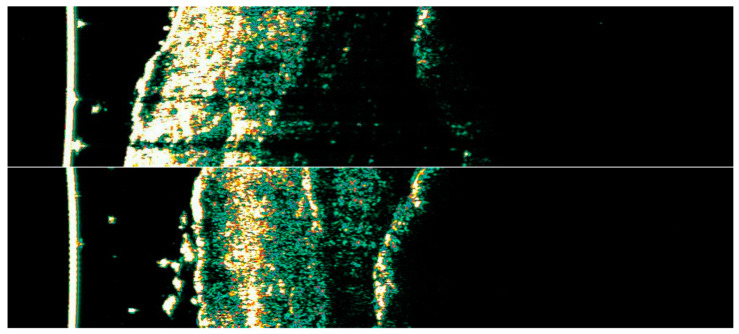
Ultrasonographic images of BCC on the temple.

**Figure 28:**
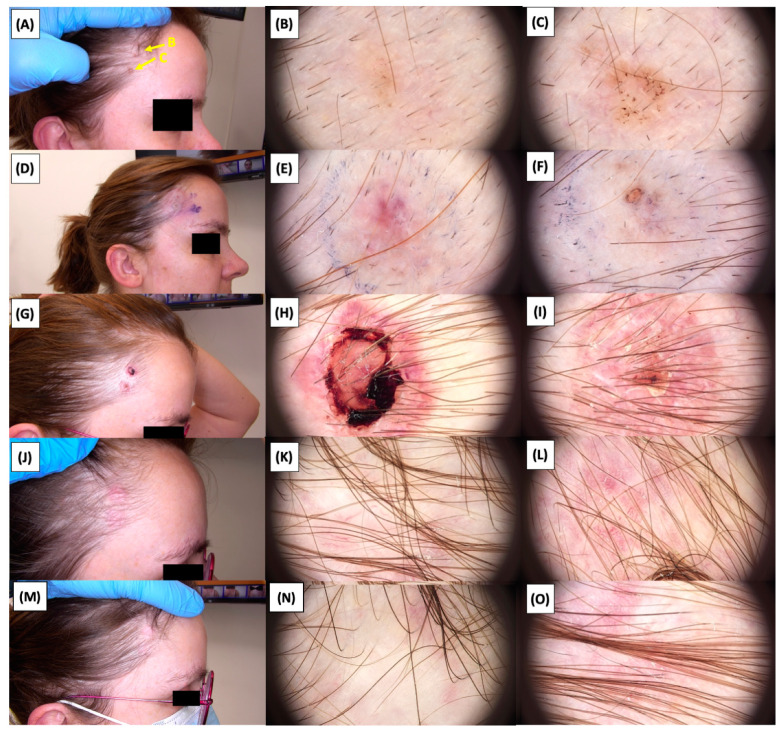
Involution of Basal Cell Carcinoma (BCC) on the temple back treated with High-Intensity Focused Ultrasound (HIFU). (**A**) Macroscopic view before HIFU treatment; (**B**) Dermoscopic view before HIFU treatment; (**C**) Dermoscopic view before HIFU treatment; (**D**) Macroscopic view immediately after HIFU treatment; (**E**) Dermoscopic view immediately after HIFU treatment; (**F**) Dermoscopic view immediately after HIFU treatment; (**G**) Macroscopic view 2 weeks post-HIFU; (**H**) Dermoscopic view 2 weeks post-HIFU; (**I**) Dermoscopic view 2 weeks post-HIFU; (**J**) Macroscopic view 3 months post-HIFU; (**K**) Dermoscopic view 3 months post-HIFU; (**L**) Dermoscopic view 3 months post-HIFU; (**M**) Macroscopic view 6 months post-HIFU; (**N**) Dermoscopic view 6 months post-HIFU; (**O**) Dermoscopic view 6 months post-HIFU. The yellow arrows indicate the area of the lesions.

**Table 1:** Clinical Parameters of High-Intensity Focused Ultrasound (HIFU) Treatment in Basal Cell Carcinoma Patients.

BCC Number	HIFU Procedure				
	Transducer Depth (mm)	Pulse Duration (ms)	Energy per Pulse (J/shot)	Number of Pulses	Treatment Time (min)
1.	0.8	150	1.3	78	20
2.	0.8	150	1.3	250	45
3.	0.8	150	1.3	170	20
4.	0.8	150	1.2	50	20
5.	1.8	150	1.3	100	30
	0.8	150	1.3	110	
6.	1.3	150	1.3	85	30
7.	0.8	150	1.3	145	40
8.	0.8	150	1.3	40	120
9.	0.8	150	1.3	70	
10	0.8	150	1.3	113	
11	0.8	150	1.3	123	
12	0.8	150	1.3	87	
13	0.8	150	1.3	88	
14	0.8	150	1.3	91	
15	0.8	150	1.3	159	

**Table 2:** Dermoscopic Evaluation Before HIFU Treatment.

Number of Changes	Vascular Patterns					Pigment Structures					Non-Pigmented Structures					
	Ambrozing Linear	Short Linear	Loop-like Linear	Bent Linear	Clod-like Linear	Segmentally Arranged	Radially Arranged Linear	Angular Linear	Small Gray-Blue Globules	Large Gray-Blue Globules	White Lines	Small White Structureless Areas	Pink Structureless Areas	White-Yellow Globules	Yellow Globule–Ulceration	Rosettes
1.	X	X	X		X						X		3			
2.	X										X		1			
3.		X	X						X							
4.		X				X			X							
5.	X	X	X								X		2			X
6.	X		X								X		2		X	
7.	X		X		X						X		1		X	
8.		X			X								1		X	
9.	X	X	X						X		X					
10.	X	X	X						X		X		1			
11.	X	X									X		2		X	
12.	X	X	X								X	X	2		X	
13.	X		X								X		2		X	X
14.	X	X	X										3			
15.	X	X	X						X		X		2			

The “X” in the table indicates that the specific dermoscopy characteristic is present. The numbers 1–3 of pink structureless areas indicate the degree of severity.

**Table 3:** Dermoscopic Evaluation Two Weeks Post-HIFU Treatment.

Number of Changes	Vascular Patterns								Pigment Structures					Non-Pigmented Structures					
	Ambrozing Linear	Short Linear	Loop-like Linear	Bent Linear	Clod-like Linear	Radially Arranged	Segmentally Arranged	Reticular	Segmentally Arranged Linear	Radially Arranged Linear	Angular Linear	Small Gray-Blue Globules	Large Gray-Blue Globules	White Lines	Small White Structureless Areas	Pink Structureless Areas	White-Yellow Globules	Orange Globule–Crust	Rosettes
1.														X		3		X	
2.	X		X				X	X						X		2			
3.						X								X				X	
4.	X						X							X		2		X	
5.	X					X								X		3	X	X	
6.	X		X				X							X	X	3		X	
7.	X	X				X									X	3		X	
8.	X		X			X									X	3			
9.						X								X	X	2			
10.						X								X	X	2		X	
11.	X				X											2		X	
12.	X						X									3		X	
13.	X						X							X				X	X
14.							X									2			X
15.						X									X	3		X	

The “X” in the table indicates that the specific dermoscopy characteristic is present. The numbers 1–3 of pink structureless areas indicate the degree of severity.

**Table 4:** Dermoscopic Evaluation 3 Months Post-HIFU Treatment.

Number of Changes	Vascular Patterns								Pigment Structures					Non-Pigmented Structures					
	Ambrozing Linear	Short Linear	Loop-like Linear	Bent Linear	Clod-like Linear	Radially Arranged	Segmentaly Arranged	Reticular	Segmentally Arranged Linear	Radially Arranged Linear	Angular Linear	Small Gray-Blue Globules	Large Gray-Blue Globules	White Lines	Small White Structureless Areas	Pink Structureless Areas	White-Yellow Globules	Orange Globule–Crust	Rosettes
1.	X						X	X						X		2			
2.	X							X						X		2			
3.	X							X								1			
4.								X								1			
5.	X							X								2			
6.	X							X								2			
7.	X							X								1			X
8.	X							X								2			
9.	X							X								2			X
10.							X							X		1			X
11.	X	X				X								X		2			
12.	X															2			
13.	X						X							X		2			
14.	X							X								2			
15.	X	X	X	X				X								1			

The “X” in the table indicates that the specific dermoscopy characteristic is present. The numbers 1–3 of pink structureless areas indicate the degree of severity.

**Table 5:** Dermoscopic Evaluation 6 Months Post-HIFU Treatment.

Number of Changes	Vascular Patterns								Pigment Structures					Non-Pigmented Structures					
	Ambrozing Linear	Short Linear	Loop-like Linear	Bent Linear	Clod-like Linear	Radially Arranged	Segmentally Arranged	Reticular	Segmentally Arranged Linear	Radially Arranged Linear	Angular Linear	Small Gray-Blue Globules	Large Gray-Blue Globules	White Lines	Small White Structureless Areas	Pink Structureless Areas	White-Yellow Globules	Orange Globule–Crust	Rosettes
1.	X	X						X						X		1			
2.	X							X						X		2			
3.	X															1			
4.								X								2			
5.								X								1			
6.						X									X	2			
7.	X															1			X
8.	X							X								2			
9.	X						X									2			
10.																1			X
11.	X														X	1			
12.	X															1			
13.	X	X														2			
14.	X							X									1		
15.	X														X	1			

The “X” in the table indicates that the specific dermoscopy characteristic is present. The numbers 1–3 of pink structureless areas indicate the degree of severity.

**Table 6:** Patient Adverse Event Tracking Post-HIFU Treatment.

Number of Lesion	Adverse Events (2–10 min Post-HIFU Treatment):								
	Transient Urticaria	Edema	Erythema	Hemorrhagic Purpura	Inflammation	Crust Formation	Hyperpigmentation	Hypopigmentation	Scarring
1.		X	X						
2.	X	X	X						
3.		X							
4.		X	X						
5.									
6.	X	X	X						
7.		X							
8.	X	X	X						
9.	X	X	X						
10.	X	X	X	X					
11.	X	X	X						
12.	X	X	X	X					
13.	X	X	X	X					
14.	X	X	X						
15.	X	X	X						
Patient	Adverse Events (2 weeks post-HIFU treatment)								
1.								X	X
2.			X			X			
3.				X	X	X			
4.			X						
5.						X			
6.						X			
7.			X			X			
8.			X			X			
9.						X			
10.									
11.									X
12.									
13.									
14.									
15.									
Patient	Adverse Events (3 months post-HIFU treatment)								
1.			X						X
2.									
3.			X						X
4.			X	X					
5.							X		X
6.			X						
7.			X						X
8.			X						X
9.									
10.									
11.									
12.									
13.									
14.									
15.									
Patient	Adverse Events (6 months post-HIFU treatment)								
1.									X
2.									
3.			X						X
4.			X						
5.									X
6.									X
7.							X	X	X
8.							X	X	X
9.									
10.									
11.									
12.									
13.									
14.									
15.									

**Table 7:** Comprehensive Evaluation of Patient Responses Following HIFU Procedure.

**Assessment of Patient Sensations after HIFU Procedure**								
**Patient**	**Intensity of Adverse Effects (0–3)**	**Pain Level during Treatment (0–10)**	**Pain Level a Few Minutes after Treatment (0–10)**	**Itching** **(0–10)**	**Comparative Method**	**Comparative Method (0–4)**	**Other Adverse Effects**	**Intensity of Adverse Effects (Total) (0–3)**
1.	Mild (1)	3	0	0	Surgical excision	Other methods cause significantly more discomfort (4)	None	Mild (1)
2.	Moderate (2)	7	2	0	Cryotherapy	Other methods cause slightly less discomfort (1)	None	Moderate (2)
3.	Mild (1)	1	0	0	Surgical excision	Other methods cause significantly more discomfort (4)	significant pain upon palpation, post-procedural sensory disturbances that persist for an extended duration	Mild (1)
4.	Mild (1)	3	0	0	Surgical excision	Other methods cause significantly more discomfort (4)	None	Mild (1)
5.	Mild (1)	3	0	0	Cryotherapy	Other methods cause slightly less discomfort (1)	None	Moderate (2)
6.	Mild (1)	3	0	0	Surgical excision	Other methods cause slightly less discomfort (1)	None	Mild (1)
7.	Mild (1)	3	1	0	Surgical excision	Other methods cause slightly less discomfort (1)	Burning sensation	Mild (1)
8.	Mild (1)	1	0	0	Cryotherapy	Other methods cause significantly more discomfort (4)	None	Mild (1)
**Assessment of patient sensations 2 weeks after HIFU treatment**								
**Patient**	**Intensity of adverse effects (0–3)**	**Assessment of the healing process (0–3)**	**Degree of pain in treated areas (0–10)**	**Degree of itching (0–10)**	**Comparative method**	**Comparative method (0–4)**	**Other adverse effects**	**Intensity of adverse effects (total)**
1.	Mild (1)	Satisfied (3)	1	0	Surgical excision	Other methods cause the same discomfort (2)	None	Mild (1)
2.	Mild (1)	Neutral (2)	3	0	Cryotherapy	Other methods cause slightly less discomfort (1)	None	Mild (1)
3.	Mild (1)	Very satisfied (4)	0	0	Surgical excision	Other methods cause significantly more discomfort (4)	None	Mild (1)
4.	Mild (1)	Very satisfied (4)	0	2	Surgical excision	Other methods cause significantly more discomfort (4)	None	Mild (1)
5.	Mild (1)	Satisfied (3)	1	1	Cryotherapy	Other methods cause slightly less discomfort (1)	None	Mild (1)
6.	Mild (1)	Satisfied (3)	0	1	Surgical excision	Other methods cause the same discomfort (2)	None	Mild (1)
7.	Mild (1)	Satisfied (3)	0	0	Surgical excision	Other methods cause slightly less discomfort (1)	None	Mild (1)
8.	Mild (1)	Very satisfied (4)	0	0	Cryotherapy	Other methods cause significantly more discomfort (4)	None	Mild (1)
**Assessment of patient sensations 3 months after HIFU treatment**								
1.	Mild (1)	Neutral (2)	0	1	Surgical excision	Other methods cause greater discomfort (3)	None	Mild (1)
2.	None (0)	Satisfied (3)	0	0	Cryotherapy	Other methods cause greater discomfort (3)	paresthesia and punctate pain at the treatment site	Moderate (2)
3.	Mild (1)	Satisfied (3)	0	1	Surgical excision	Other methods cause greater discomfort (3)	None	Mild (1)
4.	Mild (1)	Satisfied (3)	0	0	Surgical excision	Other methods cause significantly more discomfort (4)	None	Mild (1)
5.	Mild (1)	Neutral (2)	0	1	Cryotherapy	Other methods cause greater discomfort (3)	None	Moderate (2)
6.	Mild (1)	Satisfied (3)	0	0	Surgical excision	Other methods cause greater discomfort (3)	None	Mild (1)
7.	Mild (1)	Neutral (2)	0	0	Surgical excision	Other methods cause significantly more discomfort (4)	Fibrosis within the lesion	Mild (1)
8.	Mild (1)	Very satisfied (4)	0	0	Cryotherapy	Other methods cause significantly more discomfort (4)	None	None (0)
**Assessment of patient sensations 6 months after HIFU treatment**								
1.	Mild (1)	Satisfied (3)	0	0	Surgical excision	Other methods cause significantly more discomfort (4)	None	Moderate (2)
2.	Mild (1)	Very satisfied (4)	0	0	Cryotherapy	Other methods cause the same discomfort (2)	None	Mild (1)
3.	Mild (1)	Very satisfied (4)	0	0	Surgical excision	Other methods cause greater discomfort (3)	None	Mild (1)
4.	Mild (1)	Very satisfied (4)	0	0	Surgical excision	Other methods cause significantly more discomfort (4)	None	Mild (1)
5.	Mild (1)	Satisfied (3)	0	0	Cryotherapy	Other methods cause the same discomfort (2)	None	Mild (1)
6.	Mild (1)	Very satisfied (4)	0	0	Surgical excision	Other methods cause greater discomfort (3)	None	None (0)
7.	Mild (1)	Very satisfied (4)	0	0	Surgical excision	Other methods cause significantly more discomfort (4)	None	Mild (1)
8.	Mild (1)	Very satisfied (4)	0	0	Cryotherapy	Other methods cause significantly more discomfort (4)	None	Mild (1)

## Data Availability

The data is contained within the article, further inquiries can be directed to the corresponding authors.

## References

[1] Al-Qarqaz F., Marji M., Bodoor K., Almomani R., Al Gargaz W., Alshiyab D., Muhaidat J., Alqudah M. (2018). Clinical and Demographic Features of Basal Cell Carcinoma in North Jordan. J. Ski. Cancer.

[2] Fijałkowska M., Bonczar M., Jastrzębski I., Ostrowski P., Antoszewski B., Koziej M. (2023). Growth rate of basal cell carcinoma: A meta-analysis and systematic review. Adv. Dermatol. Allergol. Dermatologii Alergol..

[3] Wu S., Han J., Li W.-Q., Li T., Qureshi A.A. (2013). Basal-Cell Carcinoma Incidence and Associated Risk Factors in US Women and Men. Am. J. Epidemiol..

[4] Reinau D., Surber C., Jick S.S., Meier C.R. (2014). Epidemiology of basal cell carcinoma in the United Kingdom: Incidence, lifestyle factors, and comorbidities. Br. J. Cancer.

[5] Verkouteren J., Ramdas K., Wakkee M., Nijsten T. (2017). Epidemiology of basal cell carcinoma: Scholarly review. Br. J. Dermatol..

[6] Harken E.B.O., Fazio J. (2022). Basal Cell Carcinoma. Atlas of Dermatologic Diseases in Solid Organ Transplant Recipients.

[7] Quazi S.J., Aslam N., Saleem H., Rahman J., Khan S. (2020). Surgical Margin of Excision in Basal Cell Carcinoma: A Systematic Review of Literature. Cureus.

[8] Paoli J., Gyllencreutz J.D., Fougelberg J., Backman E.J., Modin M., Polesie S., Zaar O. (2019). Nonsurgical Options for the Treatment of Basal Cell Carcinoma. Dermatol. Pract. Concept..

[9] Trakatelli M., Morton C., Nagore E., Ulrich C., Del Marmol V., Peris K., Basset-Seguin N. (2014). Update of the European guidelines for basal cell carcinoma management. Eur. J. Dermatol..

[10] Kim J.Y.S., Kozlow J.H., Mittal B., Moyer J., Olencki T., Rodgers P., Bichakjian C., Armstrong A., Baum C., Bordeaux J.S. (2018). Guidelines of care for the management of basal cell carcinoma. J. Am. Acad. Dermatol..

[11] Naik M.P., Mehta A., Abrol S., Kumar S., Gupta V.S. (2016). Topical 5% 5-fluorouracil in the treatment of multifocal basal cell carcinoma of the face: A novel chemotherapeutic approach. Orbit.

[12] Oldfield V., Keating G.M., Perry C.M. (2005). Imiquimod: In superficial basal cell carcinoma. Am. J. Clin. Dermatol..

[13] Neumann K., Bettencourt M.S. (2016). Treatment of superficial basal cell carcinoma with ingenol mebutate gel, 0.05%. Clin. Cosmet. Investig. Dermatol..

[14] Gambini D., Passoni E., Nazzaro G., Beltramini G., Tomasello G., Ghidini M., Kuhn E., Garrone O. (2022). Basal Cell Carcinoma and Hedgehog Pathway Inhibitors: Focus on Immune Response. Front. Med..

[15] Cho M., Gordon L., Rembielak A., Woo T. (2014). Utility of radiotherapy for treatment of basal cell carcinoma: A review. Br. J. Dermatol..

[16] Lear J.T., Corner C., Dziewulski P., Fife K., Ross G.L., Varma S., Harwood C.A. (2014). Challenges and new horizons in the management of advanced basal cell carcinoma: A UK perspective. Br. J. Cancer.

[17] Copelan A., Hartman J., Chehab M., Venkatesan A.M. (2015). High-Intensity Focused Ultrasound: Current Status for Image-Guided Therapy. Semin. Interv. Radiol..

[18] Bove T., Zawada T., Serup J., Jessen A., Poli M. (2019). High-frequency (20-MHz) high-intensity focused ultrasound (HIFU) system for dermal intervention: Preclinical evaluation in skin equivalents. Ski. Res. Technol..

[19] Serup J., Bove T., Zawada T., Jessen A., Poli M. (2020). High-frequency (20 MHz) high-intensity focused ultrasound: New Treatment of actinic keratosis, basal cell carcinoma, and Kaposi sarcoma. An open-label exploratory study. Ski. Res. Technol..

[20] Calik J., Migdal M., Zawada T., Bove T. (2022). Treatment of Seborrheic Keratosis by High Frequency Focused Ultrasound—An Early Experience with 11 Consecutive Cases. Clin. Cosmet. Investig. Dermatol..

[21] Zawada T., Bove T. (2022). Strongly focused hifu transducers with simultaneous optical observation for treatment of skin at 20 MHZ. Ultrasound Med. Biol..

[22] Bove T., Zawada T., Jessen A., Poli M., Serup J. (2021). Removal of Common Warts by High-Intensity Focused Ultrasound: An Introductory Observation. Case Rep. Dermatol..

[23] Calik J., Zawada T., Bove T. (2022). Treatment of superficial benign vascular tumors by high intensity focused ultrasound: Observations in two illustrative cases. J. Cosmet. Dermatol..

[24] Smoczok M., Leonik S., Bergler-Czop B. (2022). High-intensity focused ultrasound technology as a non-surgical alternative to face lifting. Dermatol. Rev. Dermatol..

[25] Peris K., Fargnoli M.C., Kaufmann R., Arenberger P., Bastholt L., Seguin N.B., Bataille V., Brochez L., del Marmol V., Dummer R. (2023). European consensus-based interdisciplinary guideline for diagnosis and treatment of basal cell carcinoma—Update 2023. Eur. J. Cancer.

[26] Badash I., Shauly O., Lui C.G., Gould D.J., Patel K.M. (2019). Nonmelanoma Facial Skin Cancer: A Review of Diagnostic Strategies, Surgical Treatment, and Reconstructive Techniques. Clin. Med. Insights Ear Nose Throat.

[27] Wollina U., Bennewitz A., Langner D. (2014). Basal cell carcinoma of the outer nose: Overview on surgical techniques and analysis of 312 patients. J. Cutan. Aesthetic Surg..

[28] Rogalski C., Kauer F., Simon J.C., Paasch U. (2007). Meta-analysis of published data on incompletely excised basal cell carcinomas of the ear and nose with introduction of an innovative treatment strategy. JDDG J. Dtsch. Dermatol. Ges..

[29] Smith V., Walton S. (2011). Treatment of Facial Basal Cell Carcinoma: A Review. J. Ski. Cancer.

[30] Li H., Yuan S.-M., Yang M., Zha H., Li X.-R., Sun H., Duan L., Gu Y., Li A.-F., Weng Y.-G. (2016). High intensity focused ultrasound inhibits melanoma cell migration and metastasis through attenuating microRNA-21-mediated PTEN suppression. Oncotarget.

[31] Lucena S.R., Salazar N., Gracia-Cazaña T., Zamarrón A., González S., Juarranz Á., Gilaberte Y. (2015). Combined Treatments with Photodynamic Therapy for Non-Melanoma Skin Cancer. Int. J. Mol. Sci..

[32] Kaw U., Ilyas M., Bullock T., Rittwage L., Riha M., Vidimos A., Hu B., Warren C.B., Maytin E.V. (2020). A regimen to minimize pain during blue light photodynamic therapy of actinic keratoses: Bilaterally controlled, randomized trial of simultaneous versus conventional illumination. J. Am. Acad. Dermatol..

[33] Ang J.M., Bin Riaz I., Kamal M.U., Paragh G., Zeitouni N.C. (2017). Photodynamic therapy and pain: A systematic review. Photodiagnosis Photodyn. Ther..

[34] Warren C.B., Karai L.J., Vidimos A., Maytin E.V. (2009). Pain associated with aminolevulinic acid-photodynamic therapy of skin disease. J. Am. Acad. Dermatol..

[35] Ceilley R.I., Del Rosso J.Q. (2006). Current modalities and new advances in the treatment of basal cell carcinoma. Int. J. Dermatol..

[36] Siemionow M., Gatherwright J., Djohan R., Papay F. (2011). Cost analysis of conventional facial reconstruction procedures followed by face transplantation. Am. J. Transplant..

[37] Niculet E., Bobeica C., Onisor C., Gurau G., Nechita A., Radaschin D.S., Tutunaru D., Bujoreanu-Bezman L., Tatu A.L. (2023). Basal Cell Carcinoma Perineural Invasion and Suggestive Signs of Perineural Invasion—Findings and Perspectives. Life.

[38] Hill M.J., Hoegler K.M., Zhou A.E., Snow C.R., Khachemoune A. (2023). A systematic review of the incidence of basal cell carcinoma with perineural invasion: Conventional pathology versus Mohs micrographic surgery. Arch. Dermatol. Res..

